# Genicular nerve block in juvenile idiopathic arthritis: a randomized clinical trial

**DOI:** 10.1007/s10067-022-06389-4

**Published:** 2022-10-05

**Authors:** A. Radwan, S. Ohrndorf, H. Aly, M. Hamed, A. Khalifa, A. M. Elsaman

**Affiliations:** 1 Department of Rheumatology and Rehabilitation, Sohag University Hospital, Akhmim- Elsawmaa St, Sohag, 82524 Egypt; 2 Department of Rheumatology and Clinical Immunology, Charité - Universitätsmedizin Berlin, Berlin, Germany; 3 Department of Rheumatology and Rehabilitation, Al-Azhar University, Cairo, Egypt; 4 Department of Radiology, Sohag University Hospital, Sohag, Egypt

**Keywords:** Juvenile idiopathic arthritis, Genicular nerve block

## Abstract

**Objectives:**

This study aimed at evaluating the effect of genicular nerve block (GNB) in juvenile idiopathic arthritis (JIA) patients with persistent unilateral knee arthritis on pain, inflammatory parameters, function, and range of motion.

**Methods:**

A total of 104 JIA patients were diagnosed according to the International League Against Rheumatism (ILAR) criteria with persistent unilateral knee arthritis. They were allocated randomly into 2 groups: group 1 treated with GNB, while group 2 was treated with intra-articular triamcinolone (TA) only. Visual analogue scale (VAS) on pain, sonography of large joints in rheumatology (SOLAR) scoring system, and Lysholm scores were assessed at 0-, 2-, and 12-week intervals. Swelling and tenderness were clinically evaluated semi-quantitatively (0-3) at the same time intervals.

**Results:**

VAS pain, tenderness, swelling, and SOLAR grey scale (GS) and power Doppler (PD) scores were significantly reduced after 2 weeks in both groups (*p* < 0.05). This was greater in the GNB group regarding VAS and tenderness, while SOLAR and swelling were stronger reduced in TA group. After 12 weeks, all outcome measures showed lower values in the GNB group compared to TA, and this was significant regarding VAS pain. Moreover, Lysholm functional score was significantly increased in both groups at both intervals; and higher values were seen in the TA group compared to GNB after 2 weeks.

**Conclusion:**

GNB was able to control pain and improve function and inflammation of the knee joint in JIA patients. Though steroid attained better results after 2 weeks, GNB achieved an equivalent longer-term improvement after 12 weeks.

**Trial registration identifying number:**

NCT04687930.

**Table:** 

**Key Points** *• Persistent knee arthritis treatment in JIA is always challenging.* *• GNB was approved for treatment of pain in knee osteoarthritis.* *• GNB in the present study succeeded to control active knee arthritis and this effect was comparable to intra-articular steroid injection.*

## Introduction

JIA is the commonest chronic inflammatory arthritis in children. Its prevalence is 1/1000. It is characterized by a heterogeneous pattern of joint inflammation for at least 6 weeks. Age at onset is usually < 16 years. The clinical presentation, prognosis, and response to treatment are very heterogeneous. The knee, ankle and wrist are among the commonly involved joints in JIA [1]. The knee joint is involved in 40–60% of cases [2].

JIA is considered a lifelong disease with high morbidity rates. It affects children’s activity level and quality of life. This will later lead to a decrease in muscle power and an increase in osteopenia and fracture risks. The target in JIA is achieving joint remission with full function and range of motion, preventing permanent damage, and maintaining a good quality of life [3].

Chronic recurrent knee arthritis in JIA could lead to cartilage damage and persistent deformity and leg length discrepancy. Furthermore, persistent mono-articular knee arthritis despite remission is relatively common in JIA. Knee arthritis has a great impact on child mobility and quality of life in JIA [3, 4]. Repeated intra-articular steroid injection for treating knee arthritis or using large steroid doses could be harmful and can lead to significant cartilage loss and chondrocyte toxicity [5]. On several occasions, it is difficult to differentiate JIA activity from septic arthritis in the knee joint. Injecting steroids in such a condition could have a devastating effect [6]. Unlike intra-articular injection, nerve block is injected around the joint with no direct effect of the anesthetic on the cartilage. Step-up systemic treatment in mono-arthritis could be of a high cost, whereas achieving remission by local therapy is ideal in this case [4].

The integrity of nerve supply to the joint is essential to keep up the inflammatory cascade in rheumatoid arthritis. Hemiplegia may have a protective effect against the destructive effect of rheumatoid arthritis (RA). The paralyzed limb has lesser perfusion which may also suppress inflammation. It is not clear if this effect is related to impaired vascularity of the hemiplegic side or due to impaired nerve supply or both [7–9]. Even though GNB has been used for short-term control of pain in severely advanced osteoarthritis, its use in inflammatory arthritis did not attract the same courtesy [10]. Our research group has performed few clinical trials that showed encouraging results for the effect of nerve block in inflammatory arthritis [11–13].

There is cumulative evidence that consolidates the anti-inflammatory role of local anesthetics. It is known that local anesthetics can suppress different inflammatory leukocyte functions including adhesion, phagocytosis, and migration [14]. Likewise, they are involved in suppression of release of different inflammatory leukotrienes and neurotransmitters [13].

So far, our team has performed one study to evaluate the effect of GNB in adult rheumatoid arthritis. The present study is the first study to test this effect in JIA. The aim of the present clinical trial is to assess the effect of GNB in pediatric patients with JIA who had persistent unilateral knee arthritis regarding pain, range of motion, and inflammation. Furthermore, we attempted to evaluate the sustainability of this effect.

## Methods

### Patients

First, we obtained an ethical committee approval from the faculty of Medicine Al-Azhar University (0000016) and then we recruited JIA patients (no condition for disease duration) diagnosed after ILAR criteria [15], with persistent unilateral knee arthritis (for at least 3 months) aged ≥ 8 years at inclusion time. A written consent was signed from the study participants or their watchers to be included in the study and publish the materials from the collected data. Participants with severe knee destruction, ankylosed knee, peripheral neuropathy, those receiving anticoagulant therapy, skin infection, uncooperative, prior injection in the same knee in the last 6 months, or those who had an allergy to lidocaine were excluded from the study. All the participants used disease-modifying anti-rheumatic drugs and non-steroidal anti-inflammatory drugs. All the systemic medications were not changed during the study. Furthermore, all participants were informed thoroughly about the methodology, goals, and possible complications of the trial. The study was also registered on clinicaltrials.gov number NCT04687930. Medical and personal information was kept confidential. Regarding the sample size, we planned a study of a continuous response variable from matched pairs of study subjects. We calculated that we would need at least 48 pairs of subjects to be able to reject the null hypothesis that this response difference is zero with a probability (power) of 0.99. The type I error probability associated with this test of this null hypothesis is 0.95. We used the “Power and Sample Size Calculations software, version 3.1.2” for this purpose.

**Fig. 1:**
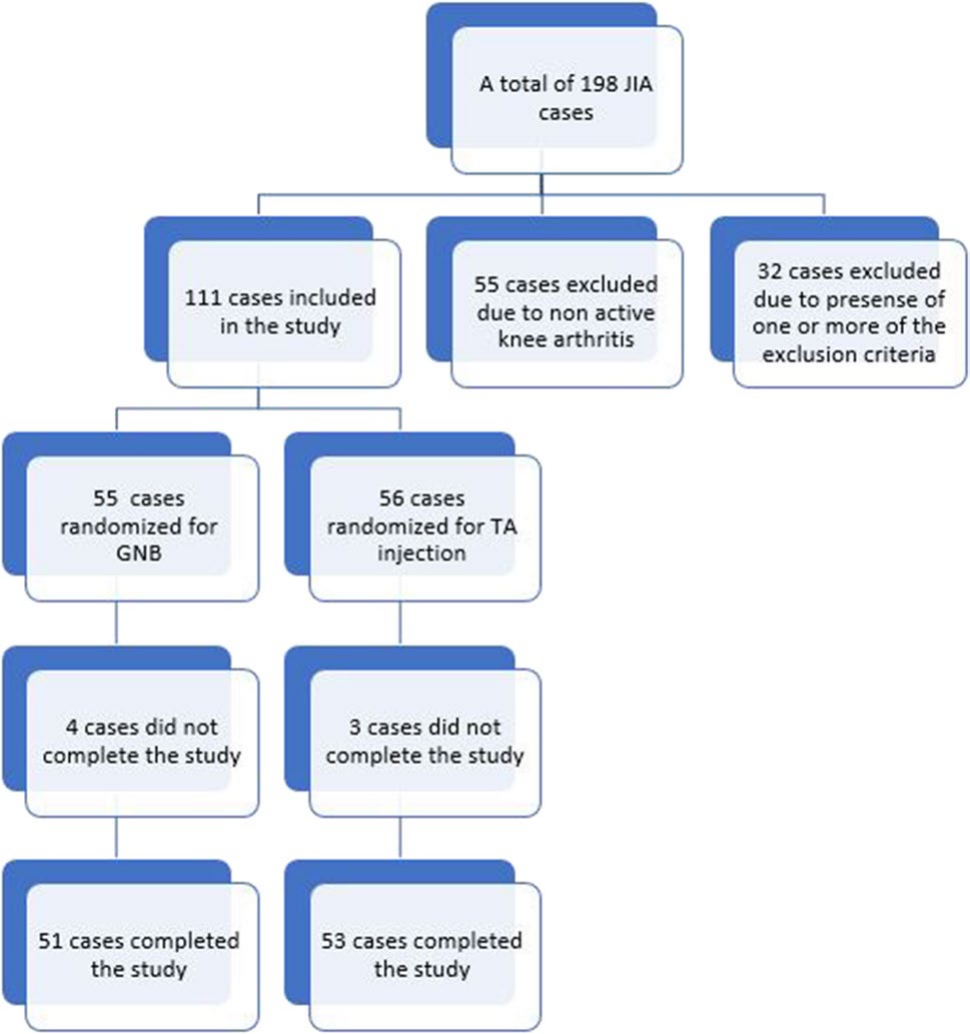
Flow chart of the included cases in the study

A total of 198 JIA cases were enrolled in the study. Out of them, 87 cases were excluded either due to the presence of one of the exclusion criteria or due to the absence of knee involvement, yielding 111 cases fulfilling the inclusion criteria for this study (see the flow chart in Fig. 1).

### Randomization and blinding

Randomization was done using the 1:1 allocation. For every two participants, the first child selected a group number from a box and the following was allocated to the opposite group. Moreover, participants were also blinded for the nature of the injected substance. Clinical evaluation, initial US evaluation, randomization, and blinding were guaranteed by the 1st author.

### Study design

Participants were enrolled from the rheumatology clinics in Al-Azhar University Faculty of Medicine. They were monitored at baseline, after 2 weeks, and 12 weeks. The different outcome measures including clinical examination, Lysholm score, SOLAR score, and VAS were assessed in each visit. Juvenile arthritis disease activity score (JADAS)-ESR was used only at baseline. Tenderness and swelling of the affected knee were also scored semi-quantitatively from 0 to 3 [16]. Participants with bilateral knee arthritis were excluded to avoid statistical errors. Likewise, those younger than 8 years were excluded as they will not be able to complete scores by themselves and intervention would be more challenging. All participants in the present study were assigned randomly into two groups: group 1 received GNB, whereas group 2 received intra-articular steroid injection. The ultrasound (US) examination and injection were conducted by two skilled sonographers. Both were blinded to clinical data.

#### GNB

Participants were asked to sit supine with a pillow supporting the popliteal fossa. The examined part was sterilized, and a 12 MHz linear probe (Toshiba Aplio 400 US system) was arranged. The transducer was turned sagittal at the front of the distal end of the femur. The probe was moved from medial or lateral to detect the femoral epicondyle. The genicular artery was identified near the periosteum and confirmed by PD. The genicular nerve is next to the artery. We targeted 3 genicular nerves: the superior medial, lateral, and inferior medial genicular nerves. The in-plane technique was considered and aspiration was done first to avoid intra-vascular injections [10]. Each nerve was injected with 2 ml of lidocaine hydrochloride 2% (Xylocaine, AstraZeneca). The vital signs were assessed twice, before and half an hour after the procedure.

### Intra-articular steroid injection

The participant was supine, with 30° knee flexion. The probe was directed axially, and the quadriceps tendon was recognized with the suprapatellar recess below. The sterile technique was considered for injection. The needle was introduced from the lateral side to medial one after infiltration anesthesia using 2 ml 1% lidocaine hydrochloride (Xylocaine, AstraZeneca). After proper setting of the needle, the injection of 1 mL of TA 40 mg (Kenacort, Bristol Myers Squibb) was done. Doppler was used to confirm the accuracy of the injection [17]. Vital signs and blood sugar were recorded before and after the procedure.

### Outcome measures

#### VAS on pain

VAS for the involved knee was also done at the same intervals. The VAS was mounted from 0 to 10. Grade 0 equals no pain and 10 signifies the worst possible pain [18–20].

#### Semi-quantitative score for swelling and tenderness

Swelling and tenderness were measured using a semi-quantitative score graded from 0 to 3; a score of 0 means no swelling nor tenderness and a score of 3 means maximum swelling and tenderness [16].

#### JADAS-ESR

JADAS includes 4 domains: physician global assessment of disease activity (0 means no activity and 10 means maximum activity), parent/patient global assessment of well-being (0 means very well and 10 means very poor), number of active joints, and an inflammatory marker ESR. We used JADAS27 version. The JADAS27 includes a selected count of the following joints: cervical spine, elbows, wrists, metacarpophalangeal joints (from first to third), proximal interphalangeal joints, hips, knees, and ankles. This is based on previous analysis that showed that the 27-joint reduced count is a good surrogate for the whole joint count in JIA [21].

#### Lysholm score

**Fig. 2:**
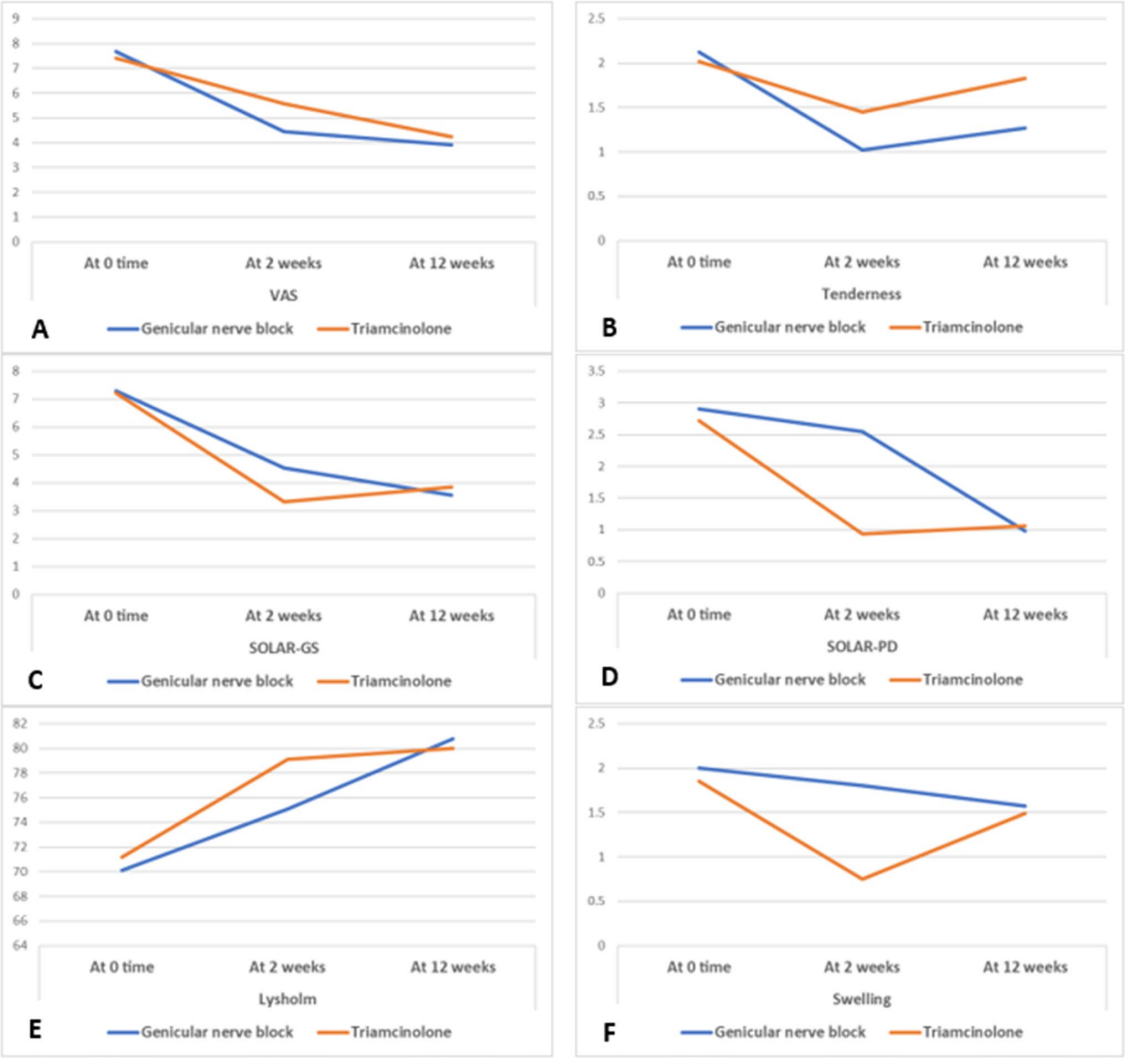
Comparison of the outcome between the two study groups: **A** VAS in the two study groups. **B** Tenderness score in the two study groups. **C** SOLAR score GS in the two study groups. **D** SOLAR score PD in the two study groups. **E** Lysholm score in the two study groups. **F** Swelling score in the two study groups

It was used for knee function assessment at 0-, 2-, and 12-week intervals. This questionnaire has 8 subsets. A score between 95 and 100 means excellent functional performance, good 84–94, fair 65–83, and poor < 64 (Fig. 2) [22]. This score was usually used for orthopedic purposes; however, the only available study that assessed the effect of GNB in RA considered this score [13]. So, we decided to use the same score for a better contrast.

#### SOLAR score

We considered the SOLAR score for sonographic evaluation of the knee in active and control groups at 0-, 2-, and 12-week intervals. A semi-quantitative 0–3 grey GS and PD scores were used. The suprapatellar midline longitudinal scan, medial longitudinal scan, lateral longitudinal scan, and posterior longitudinal scan were considered for GS, and a sagittal infrapatellar scan was added for PD score. For the GS score, the range was from 0 to 12 and for PD the score ranged from 0 to 15 [23].

### Statistical analysis

Quantitative data were expressed as means and standard deviations (SD). We used the Shapiro–Wilk test as numerical means of assessing normality of the quantitative values. Qualitative data were stated as frequencies (No.) and percentages (%). Independent sample *t*-test was used for comparing the means of the treatment arms, and Mann–Whitney test was used in cases of non-parametric distribution of data. Paired *t*-test was used to distinguish the significant differences between time points (baseline, 2 weeks, and 12 weeks). Statistical analyses were done using IBM-SPSS software program (version 25; August 2017, IBM Corporation, USA).

Regarding the sample size, we planned a study of a continuous response variable from matched pairs of study subjects, and we found that at least 48 pairs of subjects were needed to reject the null hypothesis with a probability (power) of 0.99. The type I error probability associated with this test of this null hypothesis is 0.95. We used the “Power and Sample Size Calculations software, version 3.1.2” for this purpose.

The interobserver Cohen’s kappa value (*k* value) was 0.65 at the baseline, 0.69 at 2 weeks, and 0.73 at 12 weeks, which signifies good to excellent agreement between the two observers.

## Results

The mean age (± SD) of the GNB group was 11.6 ± 2.2 years compared to 11.8 ± 2.3 years in TA group. Female percentages were slightly higher than males in the two groups (58.8%/41.2% in the GNB group and 62.3%/37.7% in the group). All of the included patients had a disease duration of more than 1-year duration. The most common type of JIA was oligoarticular (seen in around 58% of the cases), followed by polyarticular RF positive (15%), then enthesitis-related JIA (9%), undifferentiated JIA (7%), polyarticular RF negative JIA (5%), psoriatic JIA (4%), and lastly systemic JIA (3%). The mean JADAS of the studied patients was 6.67 ± 5.27 (median 5) for GNB and 6.62 ± 4.90 (median 5) for TA groups, with a non-significant difference. The prevalence of right knee affection in GNB was 64.7%, compared to 75.5% in TA group. On the other hand, the frequency of left knee involvement was 56.9% among GNB group, while it was 49.1% in TA group. Here, we calculated knee involvement whether persistent for 3 months or not, and so some cases may actually had bilateral knee involvement but had only one knee with persistent involvement. The injected knee was the right knee in 58.8% of GNB and 64.2% of TA group. No significant differences were found statistically between the two groups as regards patients’ characteristics (*p* > 0.05), as shown in Table 1.

**Table 1:** Demographic and clinical data of the study groups

Item		Group 1 (GNB)	Group 2 (TA)	*p* value
Number of cases		51 (49%)	53 (51%)	-
Age (years)		11.63 ± 2.18	11.83 ± 2.31	0.647
Sex	Male	21 (41.2%)	20 (37.7%)	0.720
	Female	30 (58.8%)	33 (62.3%)	
Disease duration (months)		19.06 ± 6.04	20.06 ± 6.83	0.432
ILAR class	sJIA	1 (2%)	2 (3.8%)	0.856
	oJIA	29 (56.9%)	31 (58.5%)	
	poJIA RF +	9 (17.6%)	7 (13.2%)	
	poJIA RF −	2 (3.9%)	3 (5.7%)	
	eJIA	5 (9.8%)	4 (7.5%)	
	pJIA	1 (2%)	3 (5.7%)	
	uJIA	4 (7.8%)	3 (5.7%)	
JADAS		6.67 ± 5.27	6.62 ± 4.90	0.965
Joints involved at baseline	Right knee	33 (64.7%)	40 (75.5%)	0.230
	Left knee	30 (58.8%)	26 (49.1%)	0.318
	Right ankle	5 (9.8%)	7 (13.2%)	0.587
	Left ankle	3 (5.9%)	6 (11.3%)	0.324
	Right wrist	10 (19.6%)	16 (30.2%)	0.213
	Left wrist	11 (21.6%)	8 (15.1%)	0.393
	Right hand	11 (21.6%)	11 (20.8%)	0.919
	Left hand	9 (17.6%)	8 (15.1%)	0.725
	Right hip	4 (7.8%)	4 (7.5%)	1.000
	Left hip	5 (9.8%)	3 (5.7%)	0.484
	Other joints	8 (15.7%)	4 (7.5%)	0.194
	Joint count	3.69 ± 3.80	3.58 ± 3.47	0.887
Joint injected	Right knee	30 (58.8%)	34 (64.2%)	0.577
	Left knee	21 (41.2%)	19 (35.8%)	

*sJIA* systemic JIA, *oJIA* oligoarticular JIA, *poJIA* polyarticular JIA, *eJIA* enthesitis-related JIA, *pJIA* psoriatic JIA, *uJIA* undifferentiated

Table 2 and Fig. 2 express the outcome measures during follow-up intervals in the studied participants. We found that tenderness, swelling, and SOLAR scores (both GS and PD) were significantly reduced 2 weeks after the interventions in the two groups (*p* < 0.05), but this significance was higher in the TA group compared to the GNB group. On the other hand, VAS score was significantly reduced 2 weeks after the interventions in both groups, but with higher significance among GNB group compared to TA group. Also, Lysholm functional score was significantly raised in the two groups 2 weeks after the intervention, with higher significance in TA group compared to GNB group (*p* < 0.05). After 12 weeks, all the outcome measures showed significant lower values in the GNB group compared to TA one. Remarkably, the GNB group revealed a longer period of decline (until 12 weeks) with a trivial rebound of the outcome measures. In the meantime, TA group displayed a faster return of the clinical and sonographic scores to the pre-intervention values after 12 weeks of interventions. Also, TA group displayed an earlier rebound of the clinical and sonographic scores to the pre-intervention values.

**Table 2:** Outcome and follow-up of the study population

Item			Group 1 (GNB)	Group 2 (TA)	*p* value
VAS	Time	At 0 time	7.67 ± 1.45	7.42 ± 1.26	0.347
		At 2 weeks	4.45 ± 1.74	5.58 ± 1.51	**0.001**
		At 12 weeks	3.90 ± 1.98	4.25 ± 1.76	0.353
	*p* values	0 vs 2 weeks	** < 0.001**	** < 0.001**	**-**
		0 vs 12 weeks	** < 0.001**	** < 0.001**	**-**
		2 vs 12 weeks	**0.014**	** < 0.001**	-
SOLAR-GS	Time	At 0 time	7.29 ± 1.98	7.23 ± 1.75	0.854
		At 2 weeks	4.53 ± 1.39	3.32 ± 1.44	** < 0.001**
		At 12 weeks	3.57 ± 1.35	3.85 ± 1.39	0.299
	*p* values	0 vs 2 weeks	** < 0.001**	** < 0.001**	**-**
		0 vs 12 weeks	** < 0.001**	** < 0.001**	**-**
		2 vs 12 weeks	** < 0.001**	**0.005**	**-**
SOLAR-PD	Time	At 0 time	2.90 ± 1.89	2.72 ± 1.66	0.596
		At 2 weeks	2.55 ± 1.08	0.94 ± 0.99	** < 0.001**
		At 12 weeks	0.98 ± 0.93	1.06 ± 0.91	0.673
	*p* values	0 vs 2 weeks	0.089	** < 0.001**	**-**
		0 vs 12 weeks	** < 0.001**	** < 0.001**	**-**
		2 vs 12 weeks	** < 0.001**	0.335	-
Lysholm	Time	At 0 time	70.1 ± 11.3	71.2 ± 11.9	0.628
		At 2 weeks	75.1 ± 9.8	79.1 ± 10.3	**0.047**
		At 12 weeks	80.8 ± 9.7	80.0 ± 10.5	0.694
	*p* values	0 vs 2 weeks	** < 0.001**	** < 0.001**	-
		0 vs 12 weeks	** < 0.001**	** < 0.001**	-
		2 vs 12 weeks	** < 0.001**	0.090	-
Tenderness	Time	At 0 time	2.12 ± 0.89	2.02 ± 0.87	0.567
		At 2 weeks	1.02 ± 0.88	1.45 ± 0.95	**0.018**
		At 12 weeks	1.27 ± 0.94	1.83 ± 0.83	**0.002**
	*p* values	0 vs 2 weeks	** < 0.001**	** < 0.001**	-
		0 vs 12 weeks	** < 0.001**	0.151	-
		2 vs 12 weeks	**0.049**	**0.005**	-
Swelling	Time	At 0 time	2.00 ± 0.94	1.85 ± 1.01	0.431
		At 2 weeks	1.80 ± 0.87	0.75 ± 0.65	** < 0.001**
		At 12 weeks	1.57 ± 0.70	1.49 ± 0.85	0.610
	*p* values	0 vs 2 weeks	**0.049**	** < 0.001**	-
		0 vs 12 weeks	**0.022**	**0.005**	-
		2 vs 12 weeks	** < 0.001**	** < 0.001**	-

Complications were found in 2% of the GNB group and 3.8% of the TA group participants, and they included pain and hematoma at the injection points.

Regarding the treatments given to the study cases, the most common DMARD used was methotrexate (received by 65.6% of the cases), followed by leflunomide (31.7%), then hydroxychloroquine (28.8%), sulfasalazine (14.5%), and lastly azathioprine (4.8%). More than half of the cases received steroids (55.8%) and most cases received NSAIDs (83.7%). None of the study cases received biological DMARDs. No significant differences were found between the two groups of the study as regards the treatment given (Table 3). Also, among the GNB cases, there were non-significant differences between those who responded well to the GNB (measured by VAS and SOLAR) and those who did not respond as regards the medical treatment given (DMARDs, steroid, or NSAIDs) as shown in Table 4.

**Table 3:** Treatment lines of the study population

Drug	Dose	GNB	TA	All cases	*p* value
Methotrexate	Non	18 (35.3%)	18 (34.0%)	36 (34.6%)	0.641 (NS)
	12.5 mg/week	22 (43.1%)	25 (47.2%)	47 (45.2%)	
	15 mg/week	10 (19.6%)	7 (13.2%)	17 (16.3%)	
	17.5 mg/week	1 (2.0%)	3 (5.7%)	4 (3.8%)	
Leflunomide	Non	31 (60.8%)	40 (75.5%)	71 (68.3%)	0.274 (NS)
	10 mg/day	14 (27.5%)	9 (17.0%)	23 (22.1%)	
	20 mg/day	6 (11.8%)	4 (7.5%)	10 (9.6%)	
Hydroxychloroquine	Non	38 (74.5%)	36 (67.9%)	74 (71.2%)	0.261 (NS)
	200 mg/day	12 (23.5%)	12 (22.6%)	24 (23.1%)	
	300 mg/day	1 (2.0%)	5 (9.4%)	6 (5.8%)	
Sulfasalazine	Non	46 (90.2%)	44 (83.0%)	90 (86.5%)	0.448 (NS)
	500 mg/day	3 (5.9%)	7 (13.2%)	10 (9.6%)	
	1000 mg/day	2 (3.9%)	2 (3.8%)	4 (3.8%)	
Azathioprine	Non	47 (92.2%)	51 (96.2%)	99 (95.2%)	0.673 (NS)
	50 mg/day	2 (3.9%)	1 (1.9%)	2 (1.9%)	
	100 mg/day	2 (3.9%)	1 (1.9%)	3 (2.9%)	
Number of DMARDs	0	0	0	0	0.927 (NS)
	1	33 (64.7%)	35 (60.0%)	68 (65.4%)	
	2	12 (23.5%)	13 (24.5%)	25 (24.0%)	
	3	6 (11.8%)	5 (9.4%)	11 (10.6%)	
Steroid	Non	23 (45.1%)	23 (43.4%)	46 (44.2%)	0.479 (NS)
	5 mg/day	15 (29.4%)	19 (35.8%)	34 (32.7%)	
	10 mg/day	8 (15.7%)	9 (7.0%)	17 (16.3%)	
	15 mg/day	2 (3.9%)	2 (3.8%)	4 (3.8%)	
	20 mg/day	3 (5.9%)	0	3 (2.9%)	
NSAIDs	Non	7 (13.7%)	10 (18.9%)	17 (16.3%)	0.478 (NS)
	Ibuprofen	40 (78.4%)	36 (67.9%)	76 (73.1%)	0.227 (NS)
	Diclofenac	4 (7.8%)	5 (9.4%)	9 (8.7%)	0.773 (NS)
	Indomethacin	0	2 (3.8%)	2 (1.9%)	0.495 (NS)

**Table 4:** Relation between good response and drug treatment among GNB group

Drug		Good responders according to VAS	*p* value	Good responders according to SOLAR	*p* value
Methotrexate	Yes	16 (48.5%)	0.782	13 (39.4%)	0.214
	No	8 (44.4%)		4 (22.2%)	
Leflunomide	Yes	8 (40.0%)	0.417	5 (25.0%)	0.311
	No	16 (51.6%)		12 (38.7%)	
Hydroxychloroquine	Yes	7 (53.8%)	0.570	4 (30.8%)	0.553
	No	17 (44.7%)		13 (34.2%)	
Sulfasalazine	Yes	1 (20.0%)	0.354	1 (20.0%)	0.454
	No	23 (50.0%)		16 (34.8%)	
Azathioprine	Yes	0	0.236	1 (33.3%)	1.000
	No	24 (50.0%)		16 (33.3%)	
Number of DMARDs	0	0	0.395	0	
	1	17 (51.5%)		8 (24.2%)	0.051
	2	6 (46.2%)		8 (61.5%)	
	3	1 (20.0%)		1 (20.0%)	
Steroid	Yes	15 (53.6%)	0.304	8 (28.6%)	0.426
	No	9 (39.1%)		9 (39.1%)	
NSAIDs	Yes	21 (47.7%)	0.568	16 (36.4%)	0.244
	No	3 (42.9%)		1 (14.3%)	

## Discussion

Persistent knee arthritis in JIA was always considered a difficult situation. In many patients, escalating systemic treatment failed to control arthritis. Local therapy was considered a practical alternative, as it can control arthritis without change in systemic treatment plans [24]. Previously, local injection of several materials comprising steroid, methotrexate, and biologics has been approved in adult rheumatoid arthritis with hopeful results [25]. GNB in JIA was not evaluated before, although it may control pain and inflammation. VAS, SOLAR score, Lysholm score, and semi-quantitative score for tenderness and swelling were used for assessment. JADAS-ESR was considered at baseline. TA outweighed GNB after 2 weeks in all parameters except for VAS and tenderness scores. After 12 weeks, the GNB group showed better results in all the outcome measures except for swelling which responded better in the steroid group and the difference was non-significant between both groups but was significant in comparison to baseline. Shorter disease duration, higher baseline SOLAR, and tenderness showed a better outcome in the GNB group after 12 weeks. Although swelling scores improved after 12 weeks, this change was non-significant for the GNB group.

It was known that GNB is effective in alleviating pain for 2 weeks on average and for function for 1 week only in osteoarthritis knee [10]. GNB effect in JIA with active knee arthritis was not tested before. In a study by Elsaman et al., GNB was effective in controlling pain, alleviating inflammation, and improving function in adult rheumatoid arthritis with persistent knee arthritis [13]. In that study, bupivacaine was used for the block. In the present study, we preferred to use lidocaine as it has a better safety profile. We used nearly the same endpoints, and the improvement was in favor of GNB after 12 weeks whereas intra-articular steroid effect was better after 2 weeks like that study. In the present study, improvement in the outcome measures for both groups was better than that in adult rheumatoid arthritis except for swelling. This effect was observed before when comparing the improvement of intra-articular steroid in JIA to adult rheumatoid arthritis [26].

In another systemic review for lower limb intra-articular in JIA, the authors found that the evidence about the role of intra-articular steroid injection is weak. This effect is more apparent in persistent oligoarticular JIA. Furthermore, the improvement was noticed in only few outcome measures. They could not reach a clear conclusion about the duration of improvement. One major obstacle they noticed is the lack of randomized clinical trials in the previous studies. They confirmed that US-guided injection is superior to blind injection with respect to accuracy and post-injection complications [2].

In another comprehensive review, Gotte noticed that more than half of the injected knees with persistent arthritis in oligoarticular JIA could achieve complete resolution of arthritis after steroid injections. The question about the duration of improvement was answered partially in this study. They recorded a mean duration of improvement of between 28 and 74 weeks. The quality, blinding, and level of evidence of the included studies were questionable. They found that good responders had higher ESR and shorter disease duration whereas the effect of sex and age at onset was controversial. Furthermore, the knee showed the longest improvement among different injected joints in some studies, and in other studies, the upper limb joints achieved a longer improvement. Regarding the complications of steroid injections, they found capsular calcification, avascular necrosis (especially for weight-bearing joints), subcutaneous atrophy with or without hypopigmentation, steroid-induced synovitis, and systemic absorption. These side effects depend on the dose, the type of injected steroid, and the frequency of the injection [27]. It is reported that JIA itself leads to decreased cartilage thickness in both upper and lower limb joints. This effect was noticed more in polyarticular and systemic types than in oligoarticular pattern. This loss may be attributed to disease activity in those joints [28].

Iversen et al. reported that patients with JIA and ankle arthritis with disturbed gait dynamics failed to return to normal gait after intra-articular steroid injection for 3 months. This was attributed to pain. This shortcoming of steroid injections highlights the necessity of treating pain in active joints in JIA especially in weight-bearing joints to restore normal gait [29].

Habib et al. reviewed the effect of intra-articular steroid injection in different diseases. They remarked that the duration of improvement depends on the type of arthritis. The duration of improvement was only 3 weeks in osteoarthritis, whereas this improvement extended to 8 weeks in adult rheumatoid and 6 months in JIA. In the listed studies in their review, the systemic treatment changes were not registered. Another essential finding is that they recorded that a single steroid injection has no effect on cartilage size [26]. In comparison, the repeated injection was associated with irreversible cartilage damage even in non-weight-bearing joints, and this damage was related to the repeated injection [30].

The elimination half-life of lidocaine is nearly 2 h [31]. It is questionable, how GNB was able to control inflammation for 3 months? The exact mechanism for this is not known and further research is needed to find out the exact mechanism. Nevertheless, it can be deduced that GNB was able to break the inflammatory cascade at a certain point and its recovery was delayed for at least 3 months.

## Limitations of the study

The small sample size is considered the chief shortcoming of the study. This can be attributed to the inclusion criteria which emphasized persistent knee arthritis. Gait analysis before and after injection should be considered in the study, but unfortunately, there is no expert for gait analysis in the university. TA hexacetanoid is better than TA acetanoide and has a longer-lasting effect. Unfortunately, TA hexacetanoid is not available in Egypt. In addition, we used a relatively small dose of TA. This can be justified by the cartilage damage noticed with using larger TA doses in previous studies [5]. Finally, it was better to extend the follow-up time to 6 months, but this would be ethically unaccepted because all systemic medications were kept unchanged during the study time.

## Conclusion

GNB could be considered a promising therapy for persistent knee arthritis in JIA patients. It can control disease activity in the knee joints based on different clinical and sonographic outcome measures. Its effect is comparable to intra-articular medications (steroid in particular) with an even better carry-on effect. It is feasible, economic, and with acceptable complications. It could limit steroid use and intensify treatment plans. This study adds more evidence that supports the anti-inflammatory effect of anesthesia. Further research is warranted to test the effect of different nerve block techniques on inflammatory arthritis.

## Data Availability

Yes.

## Abbreviations

GNB: Genicular nerve block
GS: Grey scale
ILAR: International League Against Rheumatism
JADAS: Juvenile arthritis disease activity score
JIA: Juvenile idiopathic arthritis
PD: Power Doppler
RA: Rheumatoid arthritis
SD: Standard deviation
SOLAR: Sonography of large joints in rheumatology
SPSS: Statistical Package of Social Sciences (SPSS)
TA: Triamcinolone
US: Ultrasound
VAS: Visual analogue scale
